# Total hepatic inflow occlusion vs. hemihepatic inflow occlusion for laparoscopic liver resection: a systematic review and meta-analysis

**DOI:** 10.3389/fsurg.2024.1428545

**Published:** 2024-09-26

**Authors:** Ting An, Jie Liu, Liwei Feng

**Affiliations:** Department of Pediatric Surgery, West China Hospital, Sichuan University, Chengdu, China

**Keywords:** total hepatic inflow occlusion, hemihepatic inflow occlusion, laparoscopic, liver resection, meta-analysis

## Abstract

The control of bleeding during laparoscopic liver resection (LLR) is still a focus of research. However, the advantages of the main bleeding control methods, including total hepatic inflow occlusion (TIO) vs. hemihepatic inflow occlusion (HIO), during LLR remain controversial. The purpose of this meta-analysis was to compare the clinical outcomes of patients who received TIO and patients who received HIO. This meta-analysis searched the Medline, PubMed, Web of Science, Embase, Ovid, and Cochrane Library databases. The language of the studies was restricted to English, and comparative studies of patients treated with TIO and HIO during LLR were included. The primary outcome was to compare the intraoperative details, such as the operative time, occlusion time, and volume of blood loss, between the two groups. Secondary outcomes included conversion, overall complications, liver failure, biliary leakage, ascites, pleural effusion, and hospital stay. Five studies including 667 patients, 419 (62.82%) of whom received TIO and 248 (37.18%) of whom received HIO, were included in the analysis. The demographic data, including age, sex, hemoglobin, total bilirubin, albumin, and alpha-fetoprotein, were comparable. No significant differences noted in operative time, occlusion time, volume of blood loss, conversion, overall complications, liver failure, biliary leakage, hemorrhage, ascites, or pleural effusion. The hospital stay in patients who received HIO was significantly shorter than that for patients who received TIO [mean difference (MD), 0.60; 95% confidence interval (CI), 0.33–0.87; *p* < 0.0001; *I*^2^ = 54%]. The blood loss of patients with liver cirrhosis in the TIO group was significantly less than that in the HIO group (MD, −107.63; 95% CI, −152.63 to −62.63; *p* < 0.01; *I*^2^ = 27%). Both the TIO and HIO methods are safe and feasible for LLR. Compared with HIO, TIO seems to have less blood loss in cirrhotic patients. However, this result demands further research, especially multicenter randomized controlled trials, for verification in the future.

**Systematic Review Registration:** https://www.crd.york.ac.uk/, Identifier PROSPERO (CRD42022382334).

## Introduction

With the progress of laparoscopic technology and the increase in surgeons’ learning curve, laparoscopic liver resection (LLR) has gradually evolved into a routine surgical technique (1). Some studies have reported that LLR has the advantages of faster recovery, fewer complications, and equivalent long-term outcomes compared with open procedures (2–4). However, bleeding control during LLR is still the focus and challenge of the whole operation because it is closely related to short- and long-term efficacy (5, 6). Total hepatic inflow occlusion (TIO) and hemihepatic inflow occlusion (HIO), as the two main methods of bleeding control, have been widely used in LLR (7, 8). However, the advantages of TIO vs. HIO for LLR remain controversial. The optimal approach of bleeding control during LLR needs to be evaluated with further studies, especially systematic reviews.

To the best of our knowledge, no meta-analysis has compared the efficacy of LLR treated with TIO and HIO. This meta-analysis aimed to compare the clinical outcomes of patients who underwent LLR and in the course received TIO or HIO.

## Methods

### Information source and search strategy

The results of the systematic review and meta-analysis were reported in accordance with the Preferred Reporting Items for Systematic Reviews and Meta-Analyses (PRISMA) and the Assessing the Methodological Quality of Systematic Reviews (AMSTAR) guidelines (9). This meta-analysis searched databases such as Medline, PubMed, Web of Science, Embase, Ovid, and the Cochrane Library. The search terms used were “hepatectomy”, “liver resection”, “hepatic resection”, “laparoscopy”, “laparoscopic”, “hepatic inflow occlusion”, “Pringle maneuver”, “TIO”, and “HIO”. The language was restricted to English, but the publication period was not restricted. The meta-analysis was registered in the PROSPERO database (CRD42022382334).

### Eligibility criteria

Studies that met the following criteria were included: (1) populations (P): the study involving patients who underwent LLR and received hepatic inflow occlusion performed during the operation; (2) intervention (I): the intervention mainly involves patients receiving TIO during LLR; (3) comparison (C): the comparison mainly involves patients receiving HIO during LLR; (4) outcomes (O): the outcomes included intraoperative and post-operative details such as operative time, occlusion time, blood loss, and complications; (5) study design (S): randomized controlled trials and retrospective studies were included in this analysis; and (6) the studies without full texts were excluded.

### Study selection and data collection

Two authors (TA and JL) independently screened the titles and abstracts of every article. Each step of the research selection process needed to be completely consistent. Disagreements were resolved through discussion with a third author (LF).

For each included study, the two authors (TA and JL) read the full text separately. Both researchers independently extracted relevant data especially their characteristics and outcomes. LWF reviewed all the data and arbitrated disagreements. The full extraction Excel package is displayed in Supplementary Material 1.

### Data items

The primary outcome was to compare the intraoperative details, such as the operative time, occlusion time and volume of blood loss, between the two groups. Secondary outcomes included conversion, overall complications, liver failure, biliary leakage, ascites, pleural effusion, early mortality, and hospital stay.

### Quality assessment

The quality and bias risk of the studies were evaluated according to the Newcastle‒Ottawa Quality Assessment Scale (NOS) (10). A study with a score of 1–3 was considered “low quality,” a score of 4–6 was considered “moderate quality,” and a score of 6–9 was considered “high quality.” Studies with scores above 6 were included in the meta-analysis.

### Evidence evaluation

The Grading of Recommendations, Assessment, Development, and Evaluations (GRADE) system was used to assess the certainty of evidence for critical and some important outcomes. A summary table of the GRADE evidence profile was created according to the evaluation of risk of bias, inconsistency, indirectness, imprecision, and other considerations. The evidence was divided into very low (we were very uncertain about the estimate), low (further study is very likely to have an important impact on our confidence in the estimate of effect and is likely to change the estimate), moderate (further study is likely to have an important impact on our confidence in the estimate of effect and may change the estimate), and high quality (further study is very unlikely to change our confidence in the estimate of effect).

### Statistical analysis

Review Manager 5.4 software was used for statistical analysis. Mean differences (MDs) and odds ratios (ORs) with 95% confidence intervals (CIs) were calculated to analyze continuous variables and dichotomous variables, respectively. The use of fixed- or random-effects models was based on heterogeneity. A *p*-value >0.1 and an *I*^2^ <25% suggested a lack of heterogeneity, and a fixed-effects model was used to calculate the pooled estimate, while a random-effects model was used in other cases. A *p*-value <0.1 and an *I*^2^ ranging from 25% to 50% were considered to indicate low heterogeneity. A *p*-value <0.1 and an *I*^2^ ranging from 50% to 75% were considered to indicate moderate heterogeneity. A *p*-value of the *Q*-test <0.1 and an *I*^2^ ranging from 75% to 100% were considered to indicate high heterogeneity. The data lacking standard deviation were calculated according to formulas from the Cochrane handbook throughout the study (11, 12).

## Results

### Study strategy and study selection

**Figure 1:**
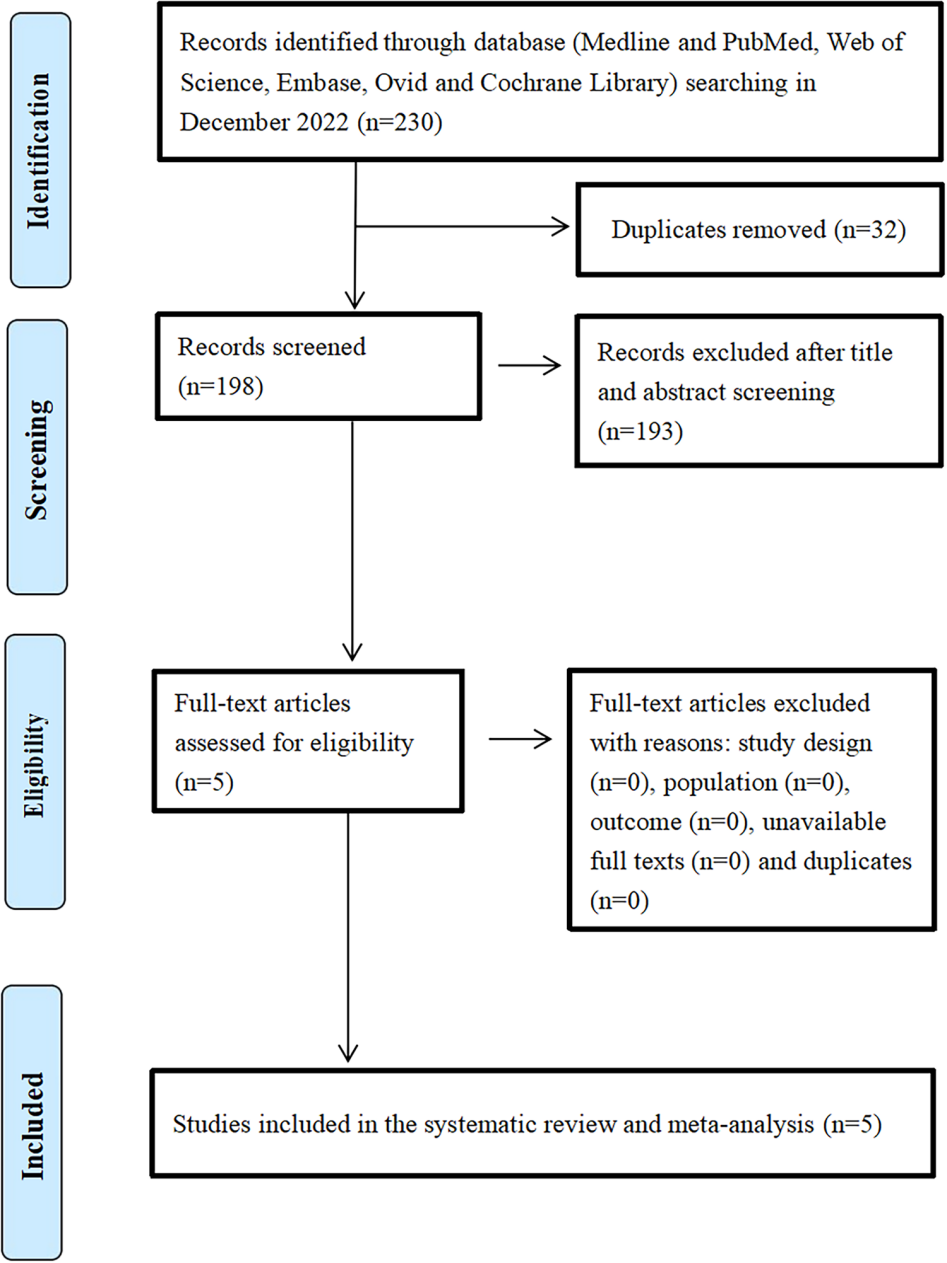
The flowchart of this systematic review and meta-analysis.

A total of 230 articles were identified through the database and other source searches, and 32 records were removed because they were duplicates (Figure 1). Subsequently, 198 articles were screened, and 193 articles were excluded due to lack of relevance after title and abstract review. Finally, five studies involving 667 patients, of which 419 (62.82%) received TIO and 248 (37.18%) received HIO, were included in the analysis (13–17).

### Study characteristics and risk of bias

The main characteristics of the patients included in the studies and the main results of the meta-analysis, including all the outcomes, are presented in Tables 1, 2. The other detailed data on patient characteristics are presented in Supplementary Table S1. The characteristics of the patients with liver cirrhosis are presented in Supplementary Table S2. The selected studies included a randomized controlled trial and four retrospective studies. The sample sizes of the included studies ranged from 30 to 258, and the publication dates ranged from 2012 to 2022. All five studies were performed in China. The Newcastle‒Ottawa Quality Assessment Scale was used to assess quality, and the results showed that the five included studies were of high quality.

**Table 1:** Demographic data of included studies.

	Year	Country	Design of study	Year of publication	Sample size		The scores based on NOS	Resection extent (major/minor)	
					TIO	HIO		TIO	HIO
Peng et al.	2022	China	Randomized clinical trial	2017–2019	129	129	8	78/51	79/50
Zhang et al.	2018	China	Retrospective study	2011–2016	16	20	6	8/8	9/11
Lan et al.	2018	China	Retrospective study	2015–2017	113	34	6	—	—
					68	24		—	—
Li et al.	2015	China	Retrospective study	2006–2015	78	26	6	—	—
Tan et al.	2012	China	Retrospective study	2004–2010	15	15	6	—	—

TIO, total hepatic inflow occlusion; HIO, hemihepatic inflow occlusion; NOS, Newcastle–Ottawa quality assessment scale; —, unknown.

**Table 2:** Main results of meta-analysis including all the outcomes.

Variable	No. of studies	No. of patients		Effect estimate	*P*-value	Heterogeneity		Effect model	
		TIO	HIO	OR/MD/HR (95% CI)		*I*^2^ (%)	*P*	Fixed	Random
Patient baseline characteristics									
Age (13–17)	5	419	248	0.29 (−1.50 to 2.08)	0.75	0	0.68	√	
Age (cirrhosis) (15, 17)	2	123	71	0.77 (−2.36 to 3.90)	0.63	0	0.53	√	
Sex, male (13–17)	5	419	248	1.38 (0.965 to 1.97)	0.08	0	0.70	√	
Sex, male (cirrhosis) (15, 17)	2	123	71	1.08 (0.47 to 2.48)	0.85	0	0.89	√	
Hemoglobin (16, 17)	2	145	149	3.64 (−1.39 to 8.67)	0.16	0	0.66	√	
Alanine transferase (14–17)	4	404	233	2.35 (−4.43 to 9.12)	0.50	95	<0.00001		√
Alanine transferase (cirrhosis) (15, 17)	2	123	71	−30.34 (−124.35 to 63.68)	0.53	87	0.006		√
Aspartic aminotransferase (14–17)	4	404	233	3.25 (−3.15 to 9.65)	0.32	95	<0.00001		√
Aspartic aminotransferase (cirrhosis) (15, 17)	2	123	71	−32.15 (−131.36 to 67.05)	0.53	84	0.01		√
Total bilirubin (14, 16, 17)	3	223	175	−0.92 (−2.02 to 0.18)	0.10	0	0.49	√	
Albumin (14, 16, 17)	3	223	175	−0.61 (−1.49 to 0.28)	0.18	0	0.61	√	
AFP (15)	1	181	58	76.53 (51.09 to 101.98)	<0.00001	0	0.82	√	
Procedure-related outcomes									
Operative time (14, 16, 17)	4	341	222	4.19 (−21.39 to 29.77)	0.75	75	0.003		√
Operative time (cirrhosis) (15, 17)	2	123	71	−28.38 (−58.56 to 1.81)	0.07	0	0.36	√	
Occlusion time (13–17)	5	419	248	1.43 (−8.12 to 10.97)	0.77	78	0.0003		√
Occlusion time (cirrhosis) (15, 17)	2	123	71	−8.26 (−21.49 to 4.97)	0.22	0	0.92	√	
Blood loss (13–17)	5	419	248	−27.63 (−87.64 to 32.39)	0.37	94	<0.00001		√
Blood loss (cirrhosis) (15, 17)	2	123	71	−107.63 (−152.63 to −62.63)	<0.00001	27	0.24		√
Conversion (16, 17)	2	145	149	1.12 (0.15 to 8.22)	0.91	0	0.91	√	
Overall complication (13–17)	5	419	248	1.24 (0.80 to 1.91)	0.34	0	0.62	√	
Overall complication (cirrhosis) (15, 17)	2	123	71	0.71 (0.35 to 1.46)	0.36	0	0.65	√	
Liver failure (13–17)	5	419	248	0.43 (0.13 to 1.42)	0.17	—	—		√
Biliary leakage (13–17)	5	419	248	0.42 (0.09 to 1.95)	0.27	0	0.73	√	
Hemorrhage (13, 14, 16, 17)	4	238	190	0.72 (0.12 to 4.41)	0.73	0	0.62	√	
Fever (13–17)	5	419	248	0.85 (0.18 to 4.02)	0.84	0	0.79	√	
Ascites (13–17)	5	419	248	0.97 (0.40 to 2.36)	0.94	23	0.27		√
Pleural effusion (13, 14, 16, 17)	4	238	190	0.88 (0.27 to 2.92)	0.84	0	0.41	√	
Drainage (14, 15)	2	259	84	13.95 (−6.66 to 34.56)	0.18	78	0.01		√
Diaphragmatic fluid infection (13–17)	5	419	248	1.01 (0.12 to 8.63)	0.99	0	0.99	√	
Early mortality (13–17)	5	419	248	1.79 (0.19 to 17.22)	0.62	0	0.62	√	
Incomplete ileus (13–17)	5	419	248	5.08 (0.24 to 106.83)	0.30	—	—		√
Hepatic insufficiency (13–17)	5	419	248	1.27 (0.07 to 21.97)	0.87	—	—		√
Infectious diarrhea (13–17)	5	419	248	3.02 (0.12 to 74.91)	0.50	—	—		√
Respiratory infection (13–17)	5	419	248	1.65 (0.73 to 3.70)	0.23	0	0.68	√	
Cough (13–17)	5	419	248	0.50 (0.04 to 5.54)	0.57	—	—		√
Wound infection (13–17)	5	419	248	0.50 (0.04 to 5.54)	0.57	—	—		√
Surgical site infections (13, 14, 16, 17)	4	238	190	1.27 (0.07 to 21.97)	0.87	—	—		√
Hospital stay (14, 15, 17)	3	388	213	0.60 (0.33 to 0.87)	<0.0001	52	0.10		√
Hospital stay (cirrhosis) (15, 17)	2	123	71	0.60 (0.35 to 0.85)	<0.00001	0	0.54	√	

TIO, total hepatic inflow occlusion; HIO, hemihepatic inflow occlusion; OR, odds ratio; HR, hazard ratio; MD, mean difference; 95% CI, 95% confidence interval; No., number.

### Analysis of demographic data

The five included studies reported demographic data of all patients or patients with liver cirrhosis, including age, sex, hemoglobin, total bilirubin (TB), albumin, and alpha-fetoprotein (AFP), and they were extremely comparable (Supplementary Figures S1, S2). No heterogeneity was found in the epidemiological data or most pre-operative examinations, while high heterogeneity was found in partial examinations, such as alanine transferase (ALT) and aspartic aminotransferase (AST) (Supplementary Figure S2).

### Analysis of outcome data

#### Operative time

**Figure 2:**
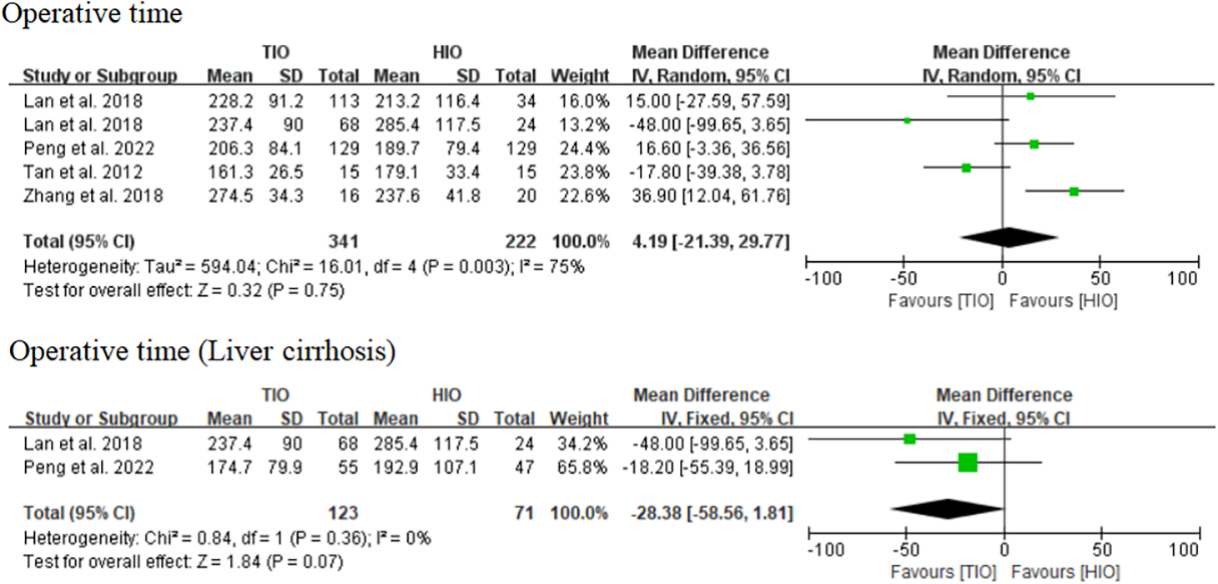
Operative time.

Four studies reported data on the operative time of TIO vs. HIO (13, 15–17), of which 341 (60.57%) patients underwent TIO and 222 (39.43%) underwent HIO. No significant difference was observed in the operative time (MD, 4.19; 95% CI, −21.39 to 29.77; *p* = 0.75) between these two groups, but the heterogeneity was high (*I*^2^ = 75%) (Figure 2). Two studies provided details on the operative time of patients with liver cirrhosis (15, 17), of which 123 (63.40%) underwent TIO and 71 (36.60%) underwent HIO. No significant difference was detected (MD, −28.38; 95% CI, −58.56 to 1.81; *p* = 0.07; *I*^2^ = 0%) between the two groups (Figure 2).

#### Occlusion time

**Figure 3:**
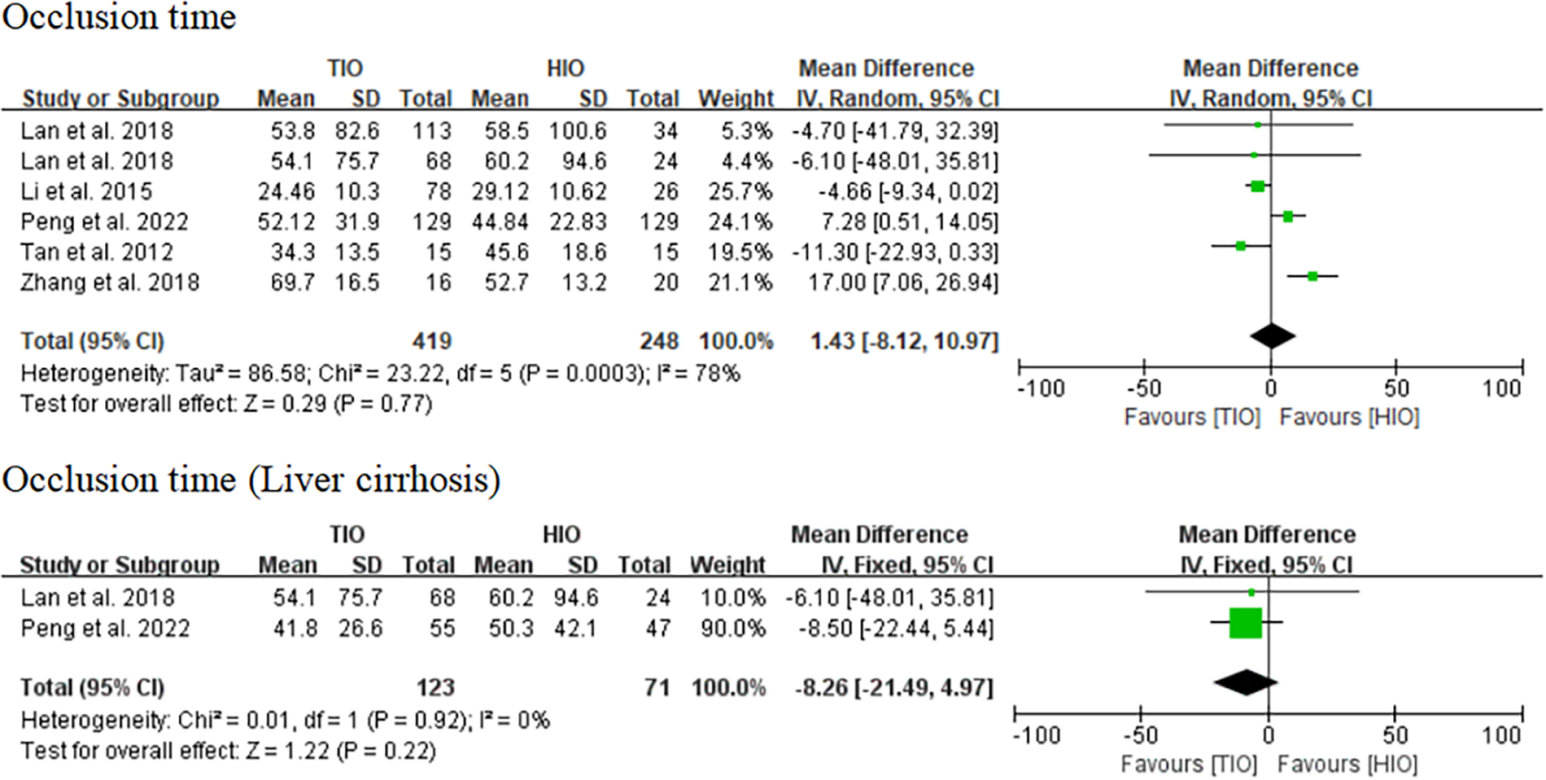
Occlusion time.

All five studies provided data on occlusion time (13–17), of which 419 (62.82%) underwent TIO and 248 (37.18%) underwent HIO. No significant difference was found in the occlusion time (MD, 1.43; 95% CI, −8.12 to 10.97; *p* = 0.77) between these two groups, but the heterogeneity was high (*I*^2^ = 78%) (Figure 3). Two studies provided information on the occlusion time of patients with liver cirrhosis (15, 17), of which 123 (63.40%) underwent TIO and 71 (36.60%) underwent HIO. There was no significant difference (MD, −8.26; 95% CI, −21.49 to 4.97; *p* = 0.22; *I*^2^ = 0%) between the two groups (Figure 3).

#### Blood loss

**Figure 4:**
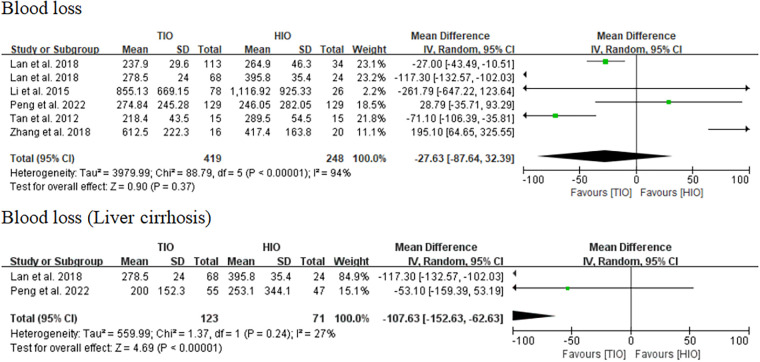
Blood loss.

All five studies provided data on blood loss (13–17), of which 419 (62.82%) involved TIO and 248 (37.18%) involved HIO. No statistically significant difference was observed in blood loss (MD, −27.63; 95% CI, −87.64 to 32.39; *p* = 0.37) between these two groups, but the heterogeneity was high (*I*^2^ = 94%) (Figure 4). Two studies offered details on the blood loss of patients with liver cirrhosis (15, 17), of which 123 (63.40%) underwent TIO and 71 (36.60%) underwent HIO. The blood loss of patients with liver cirrhosis in the TIO group was significantly lower than that in the HIO group (MD, −107.63; 95% CI, −152.63 to −62.63; *p* < 0.00001), and the heterogeneity was very low (*I*^2^ = 27%) (Figure 4). Blood loss favored TIO.

#### Conversion

**Figure 5:**
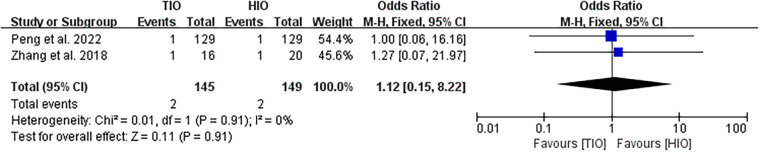
Conversion.

Two studies provided data on conversion (16, 17). Conversion occurred in 2 (1.38%) of the 145 patients treated with TIO and 2 (1.34%) of the 149 patients treated with HIO. No significant difference was found between the two groups (OR, 1.12; 95% CI, 0.15–8.22; *p* = 0.91; *I*^2^ = 0%) (Figure 5).

#### Overall complication

**Figure 6:**
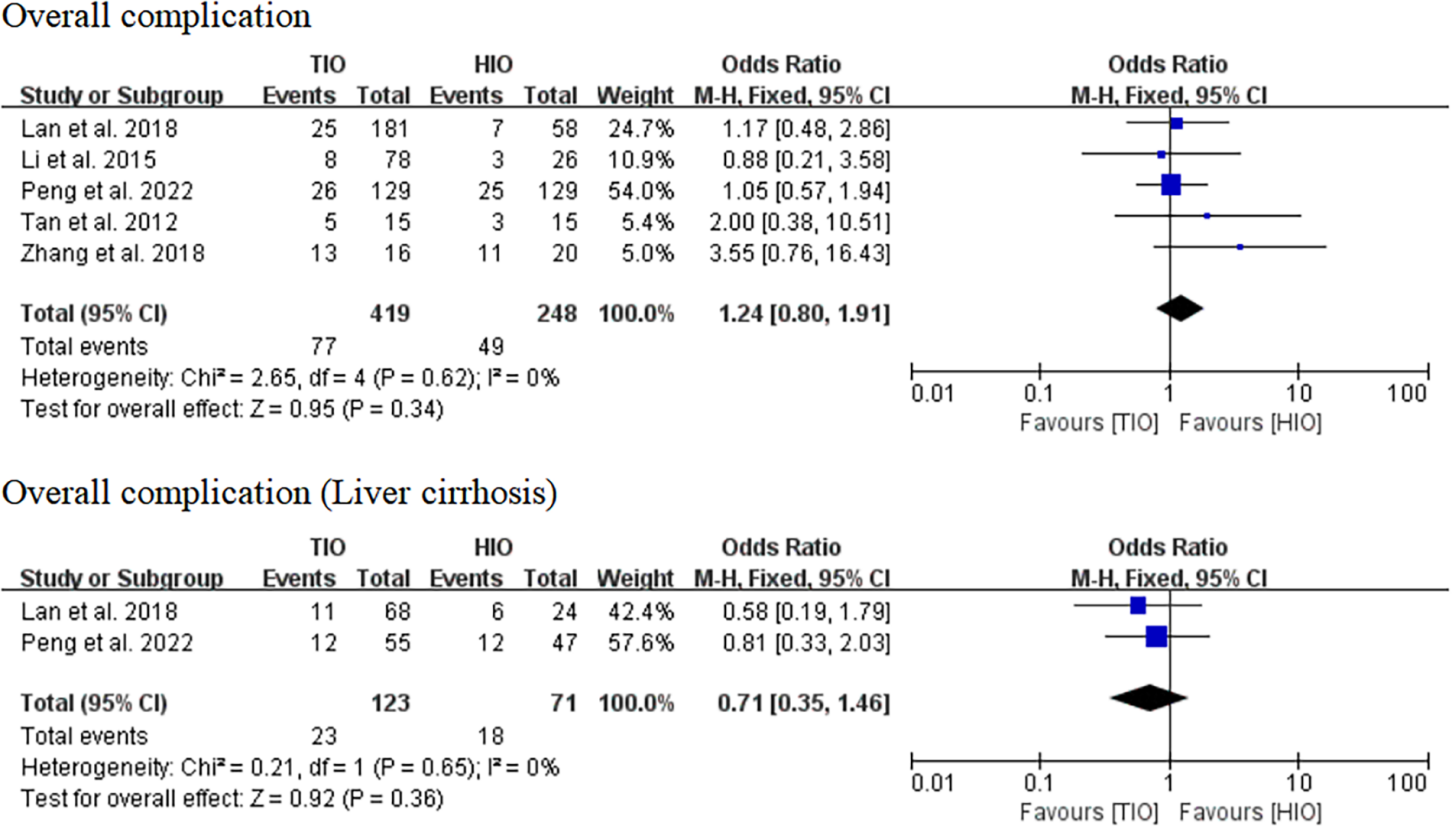
Overall complication.

All five studies provided data on complications (13–17). Overall complications occurred in 77 (18.38%) of the 419 patients treated with TIO and 49 (19.76%) of the 248 patients treated with HIO. There was no significant difference between the two groups (OR, 1.24; 95% CI, 0.80–1.91; *p* = 0.34; *I*^2^ = 0%) (Figure 6). Two studies provided details on the complications of patients with liver cirrhosis (15, 17). Overall, complications occurred in 23 (18.70%) of the 123 patients with liver cirrhosis in the TIO group and 18 (25.35%) of the 71 patients with liver cirrhosis in the HIO group. There was no difference between the two groups (OR, 0.71; 95% CI, 0.35–1.46; *p* = 0.36; *I*^2^ = 0%) (Figure 6).

#### Specific complications

**Figure 7:**
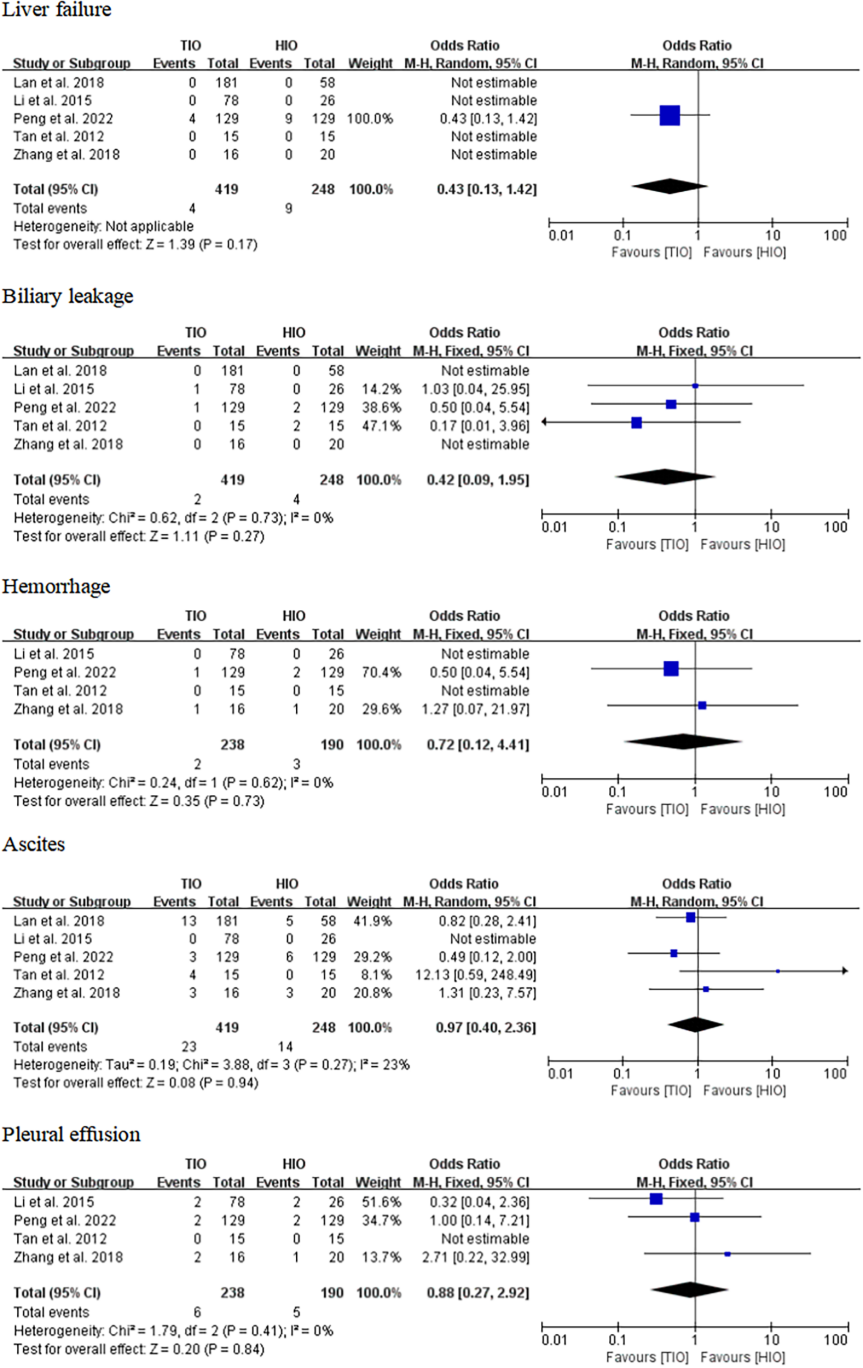
Specific complications.

Some specific complications, including liver failure, biliary leakage, hemorrhage, ascites, and pleural effusion, have been reported. Five studies offered details on liver failure, which occurred in 4 (0.95%) of the 419 patients with TIO and 9 (3.63%) of the 248 patients with HIO. There was no difference in liver failure between the two groups (OR, 0.43; 95% CI, 0.13–1.42; *p* = 0.17) (Figure 7). Five studies provided information on biliary leakage, which occurred in 2 (0.48%) of the 419 patients with TIO and 4 (1.61%) of the 248 patients with HIO. No difference was found in biliary leakage between the two groups (OR, 0.42; 95% CI, 0.09–1.95; *p* = 0.79; *I*^2^ = 0%) (Figure 7). Four studies offered details on hemorrhage, which occurred in 2 (0.84%) of the 238 patients with TIO and 3 (1.58%) of the 190 patients with HIO. There was no difference in hemorrhage between the two groups (OR, 0.72; 95% CI, 0.12–4.41; *p* = 0.73; *I*^2^ = 0%) (Figure 7). Five studies offered details on ascites, which occurred in 23 (5.49%) of the 419 patients with TIO and 14 (5.65%) of the 248 patients with HIO. There was no difference in ascites between the two groups (OR, 0.97; 95% CI, 0.40–2.36; *p* = 0.94; *I*^2^ = 23%) (Figure 7). Four studies provided information on pleural effusion, which occurred in 6 (2.52%) of the 238 patients with TIO and 5 (2.63%) of the 190 patients with HIO. There was no difference in pleural effusion between the two groups (OR, 0.88; 95% CI, 0.27–2.92; *p* = 0.84; *I*^2^ = 0%) (Figure 7).

#### Other complications

Other complications including fever, drainage, diaphragmatic fluid infection, early mortality, incomplete ileus, hepatic insufficiency, infectious diarrhea, respiratory infection, cough, wound infection, and surgical site infections were reported. No differences were found in the related complications between the two groups (Supplementary Figure S3).

#### Hospital stay

**Figure 8:**
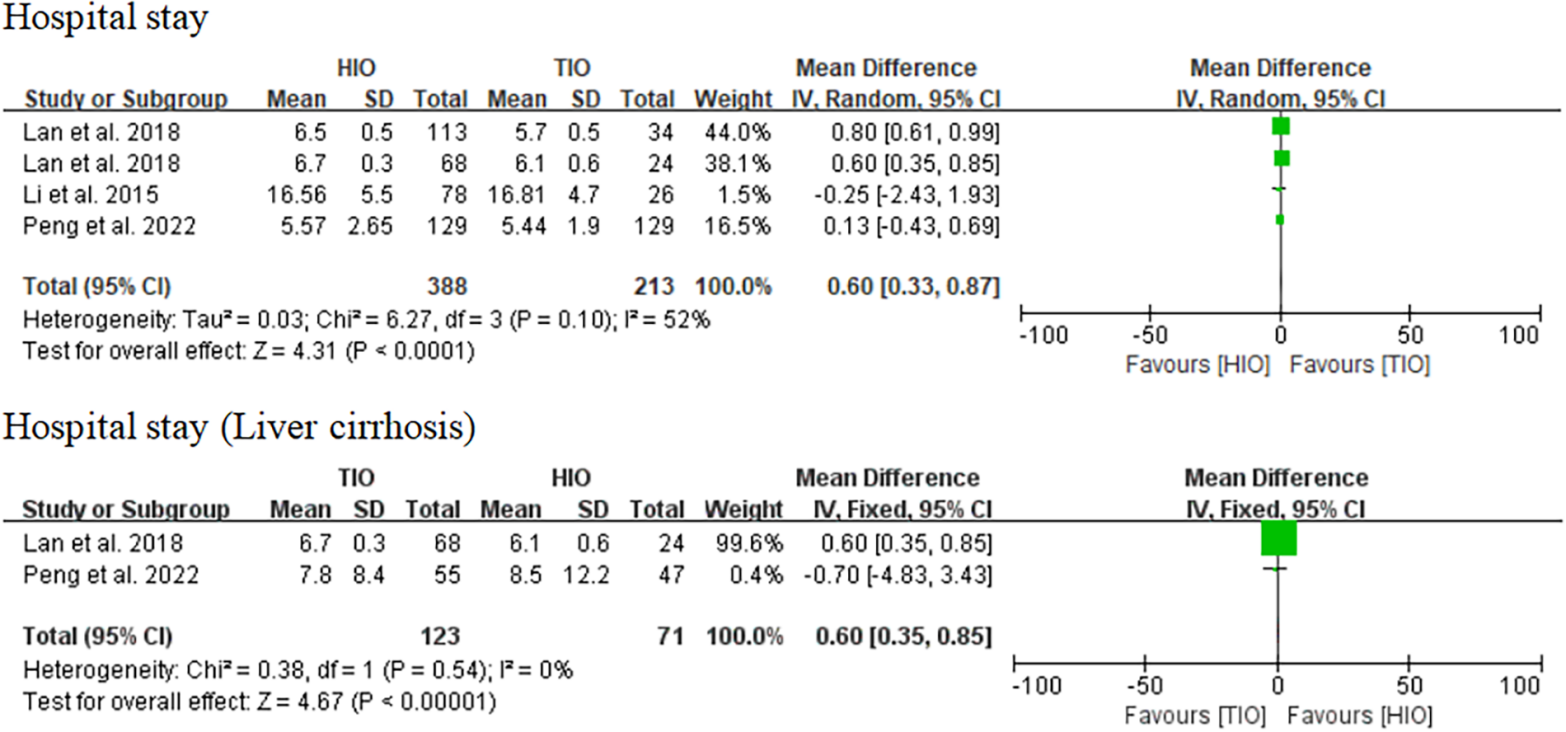
Hospital stay.

Four studies provided details on hospital stay (14, 15, 17), and the hospital stay in patients who received HIO was significantly shorter than that in patients who received TIO (MD, 0.60; 95% CI, 0.33–0.87; *p* < 0.0001), but there was moderate heterogeneity (*I*^2^ = 54%) (Figure 8). Two studies offered details on the hospital stay of patients with liver cirrhosis (15, 17), of which 123 (63.40%) underwent TIO and 71 (36.60%) underwent HIO. The hospital stay of patients with liver cirrhosis in the HIO group was shorter than that of the patients in the TIO group (MD, 0.60; 95% CI, 0.35–0.85; *p* < 0.00001; *I*^2^ = 0%) (Figure 8).

#### Post-operative liver function

Liver function parameters, such as ALT, AST, TB, and albumin, after surgery were not significantly different, and the heterogeneity was high between the two groups (Supplementary Figure S4).

#### Evidence evaluation

The GRADE evidence for critical and some important outcomes was assessed and is presented in Table 3. The level of evidence was low for operative time (liver cirrhosis), occlusion time (liver cirrhosis), blood loss (liver cirrhosis), conversion, overall complications, overall complications (liver cirrhosis), liver failure, biliary leakage, hemorrhage, ascites, pleural effusion, and hospital stay (liver cirrhosis) and very low for operative time, occlusion time, blood loss, and hospital stay.

**Table 3:** GRADE evidence for the critical and some important outcomes.

Quality assessment							No. of patients		Effect		Quality	Importance
No. of studies	Study design	Risk of bias	Inconsistency	Indirectness	Imprecision	Other considerations	TIO	HIO	Relative (95% CI)	Absolute (95% CI)		
Operative time												
4	Observational studies	Very serious^a^	No serious	No serious	No serious	None	341	222	—	MD 4.19 higher (21.39 lower to 29.77 higher)	⊕OOO Very low	Critical
Operative time (liver cirrhosis)												
2	Observational studies	No serious	No serious	No serious	No serious	None	123	71	—	MD 28.38 lower (58.56 lower to 1.81 higher)	⊕⊕OO Low	Critical
Occlusion time												
5	Observational studies	Serious^a^	No serious	No serious	No serious	None	419	248	—	MD 1.43 higher (8.12 lower to 10.97 higher)	⊕OOO Very low	Critical
Occlusion time (liver cirrhosis)												
2	Observational studies	No serious	No serious	No serious	No serious	None	123	71	—	MD 8.26 lower (21.49 lower to 4.97 higher)	⊕⊕OO Low	Critical
Blood loss												
5	Observational studies	Serious^a^	No serious	No serious	No serious	None	419	248	—	MD 27.63 lower (87.64 lower to 32.39 higher)	⊕OOO Very low	Critical
Blood loss (liver cirrhosis)												
2	Observational studies	No serious	No serious	No serious	No serious	None	123	71	—	MD 107.63 lower (152.63–62.63 lower)	⊕⊕OO Low	Critical
Conversion												
2	Observational studies	No serious	No serious	No serious	No serious	None	2/145 (1.4%)	2/149 (1.3%)	OR 1.12 (0.15–8.22)	2 more per 1,000 (from 11 fewer to 87 more) 3 more per 1,000 (from 25 fewer to 168 more)	⊕⊕OO Low	Important
Overall complication												
5	Observational studies	No serious	No serious	No serious	No serious	None	77/419 (18.4%)	49/248 (19.8%)	OR 1.24 (0.8–1.91)	36 more per 1,000 (from 33 fewer to 122 more) 36 more per 1,000 (from 33 fewer to 121 more)	⊕⊕OO Low	Critical
Overall complication (liver cirrhosis)												
2	Observational studies	No serious	No serious	No serious	No serious	None	23/123 (18.7%)	18/71 (25.4%)	OR 0.71 (0.35–1.46)	59 fewer per 1,000 (from 147 fewer to 78 more) 59 fewer per 1,000 (from 147 fewer to 78 more)	⊕⊕OO Low	Critical
Liver failure												
5	Observational studies	No serious	No serious	No serious	No serious	none	4/419 (1%)	9/248 (3.6%)	OR 0.43 (0.13–1.42)	20 fewer per 1,000 (from 31 fewer to 14 more)-	⊕⊕OO Low	Critical
Biliary leakage												
5	Observational studies	No serious	No serious	No serious	No serious	None	2/419 (0.5%)	4/248 (1.6%)	OR 0.42 (0.09–1.95)	9 fewer per 1,000 (from 15 fewer to 15 more)-	⊕⊕OO Low	Critical
Hemorrhage												
4	Observational studies	No serious	No serious	No serious	No serious	None	2/238 (0.8%)	3/190 (1.6%)	OR 0.72 (0.12–4.41)	4 fewer per 1,000 (from 14 fewer to 50 more) 2 fewer per 1,000 (from 7 fewer to 26 more)	⊕⊕OO Low	Critical
Ascites												
—	Observational studies	No serious	No serious	No serious	No serious	None	23/419 (5.5%)	14/248 (5.6%)	OR 0.97 (0.4–2.36)	2 fewer per 1,000 (from 33 fewer to 67 more) 1 fewer per 1,000 (from 28 fewer to 57 more)	⊕⊕OO Low	Critical
Pleural effusion												
4	Observational studies	No serious	No serious	No serious	No serious	None	6/238 (2.5%)	5/190 (2.6%)	OR 0.88 (0.27–2.92)	3 fewer per 1,000 (from 19 fewer to 47 more) 4 fewer per 1,000 (from 24 fewer to 58 more)	⊕⊕OO Low	Critical
Hospital stay												
3	Observational studies	Serious^a^	No serious	No serious	No serious	None	388	213	—	MD 0.6 higher (0.33–0.87 higher)	⊕OOO very low	Important
Hospital stay (Liver cirrhosis)												
2	Observational studies	No serious	No serious	No serious	No serious	None	123	71	—	MD 0.6 higher (0.35–0.85 higher)	⊕⊕OO Low	Important

GRADE, grading of recommendations assessment, development, and evaluation; TIO, total hepatic inflow occlusion; HIO, hemihepatic inflow occlusion; OR, odds ratio; MD, mean difference; 95% CI, 95% confidence interval; No, number.

^a^ Heterogeneity (*I*^2^ > 50%, *P* < 0.1) was found.

## Discussion

Our meta-analysis included a total of 667 participants from five studies. The results revealed no significant differences in perioperative details, such as operative time, occlusion time, blood loss, conversion, or overall complications, between the TIO and HIO groups during LLR. However, the blood loss of patients with liver cirrhosis in the TIO group was significantly less than that in the HIO group.

Although the LLR is becoming more and more mature, intraoperative bleeding control has always been the key and difficult point of this technology. Since Pringle and Bismuth reported the feasibility and safety of TIO (also known as the Pringle maneuver) and HIO in 1908 and 1982, respectively, these two methods have become the main means of controlling intraoperative bleeding (18, 19). However, the two methods for controlling bleeding during LLR are still controversial. Some authors believe that TIO is always a concern of ischemic injury to the residual liver, especially for those with liver cirrhosis (20). It has been reported that in East Asia, 80% of patients who undergo hepatectomy have liver cirrhosis and poor tolerance to ischemia (21). By contrast, other experts believe that the operation of HIO is technically needed. Some small communication branches still exist between the lobes of the liver; hence, there is no absolute boundary of the liver blood flow basin between the lobes. When the liver parenchyma is transected in the middle plane, HIO does not block the blood flow enough, and the relatively poor bleeding control during the operation leads to increased bleeding or unclear vision compared with TIO (22). Second, HIO still involves blood flow from the portal vein to the hepatic vein through the contralateral liver. The middle hepatic vein (MHV) is relatively more congested and the pressure increases, leading to a greater risk of bleeding, especially for the MHV, which needs to be exposed during LLR (23). HIO also increases the pressure of the inferior vena cava (IVC), and the blood flow back to the portal vein system increases the risk of bleeding. At present, large-sample data and multicenter comparative studies on TIO and HIO are still lacking. Therefore, we conducted this meta-analysis and aimed to compare the clinical efficacy of TIO and HIO during LLR.

In this meta-analysis, we found no significant differences in intraoperative data, including operative time, occlusion time, blood loss, or conversion, between the TIO and HIO. In addition, no significant differences were detected in the post-operative details, including overall complications, liver failure, biliary leakage, hemorrhage, ascites, or pleural effusion, which showed that TIO and HIO had the same clinical efficacy. In the subgroup analysis of patients with liver cirrhosis, we found that there were no significant differences in the operation time, occlusion time, and overall complications, but the volume of bleeding in the TIO group was less than that in the HIO group. This indicated that TIO was more recommended for those with liver cirrhosis during LLR. While there was no statistically significant difference in the overall incidence of complications between the two types of obstruction, it is important to emphasize the discussion of complications in laparoscopic surgery. Mazzotta et al. found that the conditional cumulative incidence of treatment-requiring complications in patients undergoing laparoscopic liver resection is effectively stratified by the three-level complexity classification. Analysis of complication risk based on these complexity grades may be helpful in optimizing in-hospital observation after laparoscopic liver resection (24).

This meta-analysis also revealed that the hospital stay of patients in the TIO group was longer than that of patients in the HIO group, including patients with liver cirrhosis. Whether this was because of the ischemic effect of TIO on the residual liver, leading to an increase in the recovery time of liver function injury after surgery, remains unclear. Although no differences in post-operative liver function were detected in our study, there was high heterogeneity. However, Peng et al. conducted a randomized controlled study in 2021 and showed that the liver functions of patients with cirrhosis after TIO were comparable to those after HIO (17). However, this study did find that the hospitalization time for HIO was shorter than for TIO, regardless of whether the patient had liver cirrhosis or not. This is an advantage of HIO over TIO.

Extreme heterogeneity of some results, such as operative time, occlusion time, blood loss, and hospital stay, was found in the study. In addition to the subgroup statistical analysis of patients with liver cirrhosis, we also tried to conduct other subgroup analyses and meta-regression analyses during the initial statistical process. However, it was regrettable that the expected results were not satisfactory owing to the scarcity and different types of current research. We attempted to remove one or two studies during the re-analysis process in an effort to reduce the heterogeneity of the results. However, sensitivity analysis showed that this did not decrease the heterogeneity. Although all five studies performed laparoscopic liver resection, only two studies provided a detailed proportion of specific surgical methods including major hepatectomy and minor hepatectomy (e.g., segmentectomy), and the other three studies did not provide detailed information. The specific surgical methods used might have differed among the five studies, which might be one of the reasons for the high heterogeneity of some of the results. A minor hepatectomy cannot be used as an exclusion criterion, as it requires a high level of technical proficiency. For example, the operative time, occlusion time, and blood loss of laparoscopic-assisted V + VI/VI + VII/V + VI + VII segments’ resection were definitely different from those of laparoscopic-assisted hemihepatectomy. This was one of the limitations of this study. The other reason for the heterogeneity differences may be the incomplete uniformity of unit surgical standards in the five articles. The reason for the lack/low heterogeneity of the liver cirrhosis subgroup analysis might be that there was no difference or small difference in the specific surgical methods or surgical procedures between the two studies.

There are several limitations in this systematic review and meta-analysis. First, not all enrolled studies were randomized controlled studies. Second, regarding operative time, it was challenging to demonstrate a significant difference, and the study may be limited by procedural bias. Third, only two articles involved patients with liver cirrhosis. Although TIO offers the advantage of less blood loss than HIO in patients with liver cirrhosis according to our study, more studies, especially multicenter randomized controlled trials, are needed to verify these results and improve the quality of evidence in the future. Fourth, other meta-analyses, such as those on operative time (liver cirrhosis), occlusion time (liver cirrhosis), conversion, overall complications (liver cirrhosis), and hospital stay (liver cirrhosis), were also based on the data of only two studies. Fifth, the power of the analyses of some rare results, such as liver failure, in this study was insufficient, and more studies with large sample sizes are needed for verification. However, to our knowledge, this is the first meta-analysis to compare LLR in patients with TIO and HIO in terms of clinical outcomes.

## Conclusion

Both the TIO and HIO methods are safe and feasible when performed during LLR. After analyzing the subgroup of patients with liver cirrhosis involved in two studies, TIO seems to have less blood loss compared with HIO; however, further studies are needed, especially multicenter randomized controlled trials, to verify this result in the future.

## Data Availability

The original contributions presented in the study are included in the article/Supplementary Material, further inquiries can be directed to the corresponding author.
