# Supplementary figures and images for: Total hepatic inflow occlusion vs. hemihepatic inflow occlusion for laparoscopic liver resection: a systematic review and meta-analysis
Source: Front Surg. 2024 Sep 26;11:1428545. doi: 10.3389/fsurg.2024.1428545 (PMC11467754; doi:10.3389/fsurg.2024.1428545)

Age
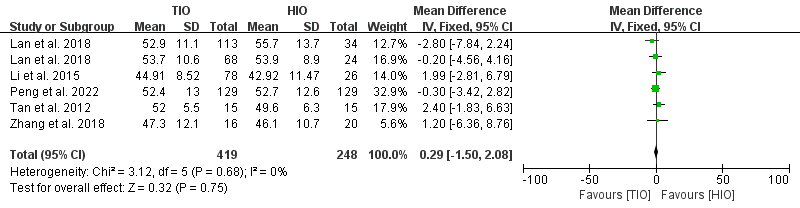


Age (cirrhosis)


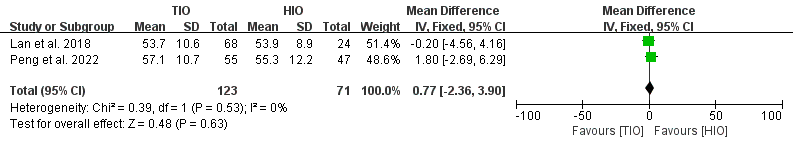


Sex


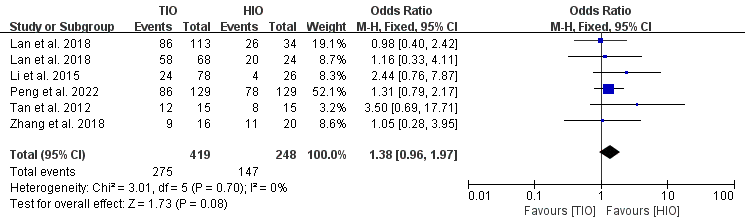


Sex (cirrhosis)


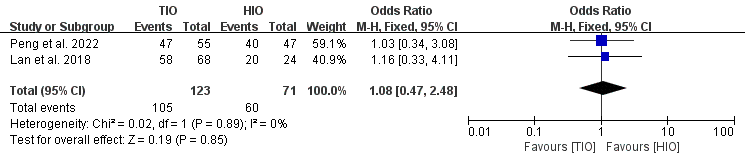

Supplement: Supplementary file 1 [file Supplementaryfile1.docx]
