# Supplementary material for: Total hepatic inflow occlusion vs. hemihepatic inflow occlusion for laparoscopic liver resection: a systematic review and meta-analysis
Source: Front Surg. 2024 Sep 26;11:1428545. doi: 10.3389/fsurg.2024.1428545 (PMC11467754; doi:10.3389/fsurg.2024.1428545)

Hemoglobin


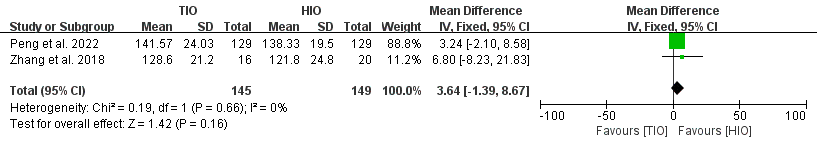


Total bilirubin (TB)


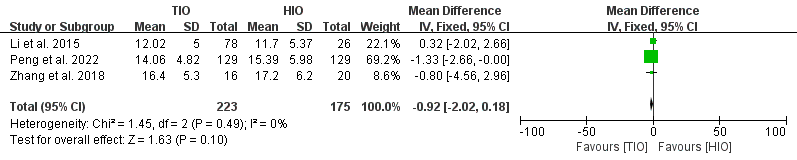


Albumin


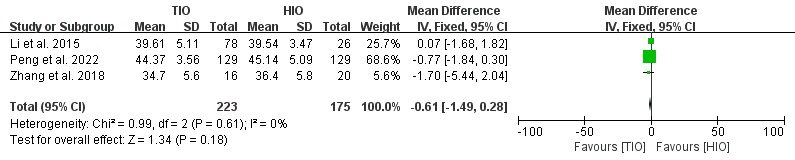


AFP


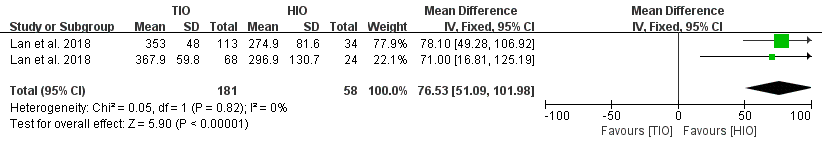


Alanine transferase (ALT)


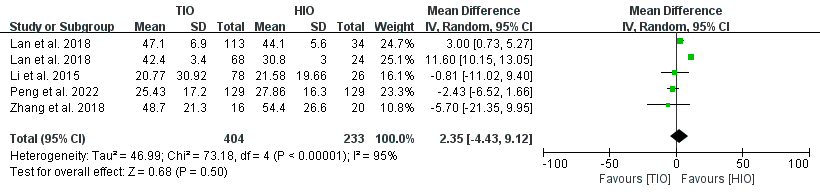


Alanine transferase (ALT) (cirrhosis)


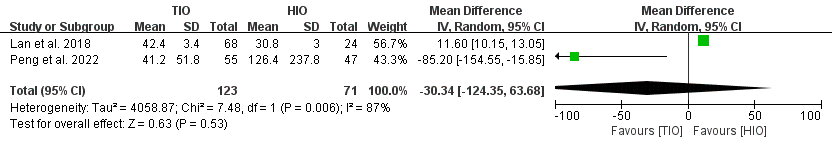


Aspartic aminotransferase (AST)


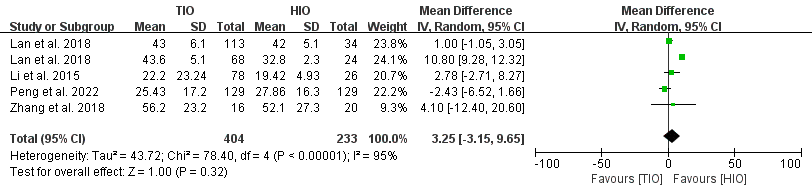


Aspartic aminotransferase (AST) (cirrhosis)


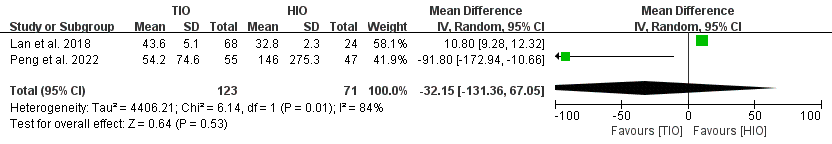

Supplement: Supplementary file 2 [file Supplementaryfile2.docx]
