# Supplementary material for: Total hepatic inflow occlusion vs. hemihepatic inflow occlusion for laparoscopic liver resection: a systematic review and meta-analysis
Source: Front Surg. 2024 Sep 26;11:1428545. doi: 10.3389/fsurg.2024.1428545 (PMC11467754; doi:10.3389/fsurg.2024.1428545)

Fever


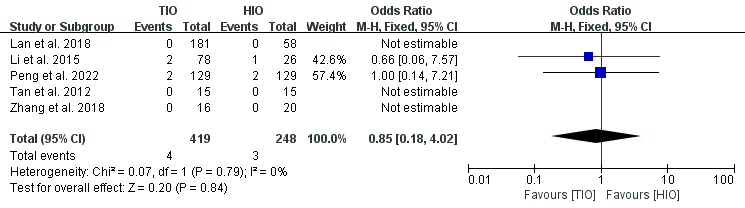


Drainage


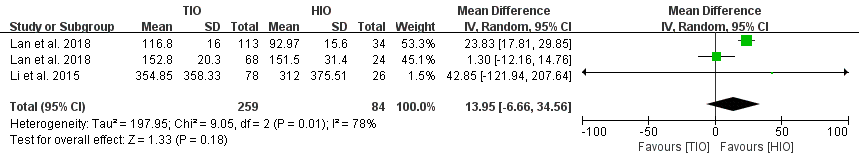


Diaphragmatic fluid infection


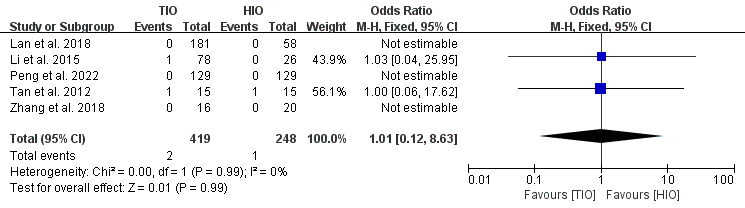


Early mortality


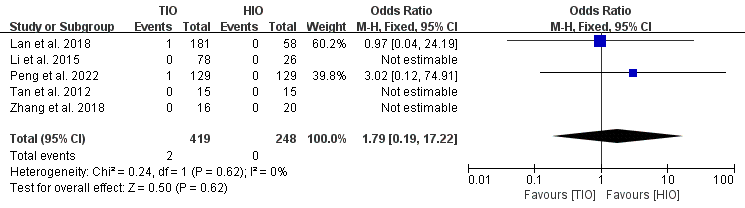


Incomplete ileus


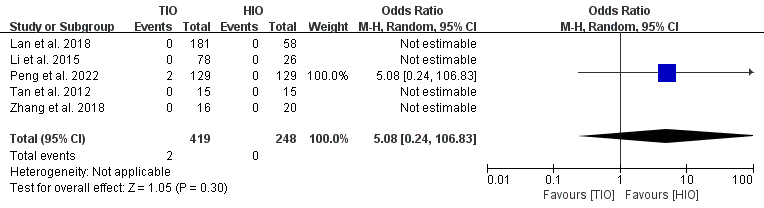


Hepatic insufficiency

**
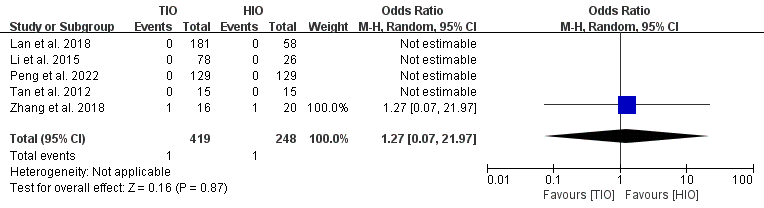
**

Infectious diarrhea


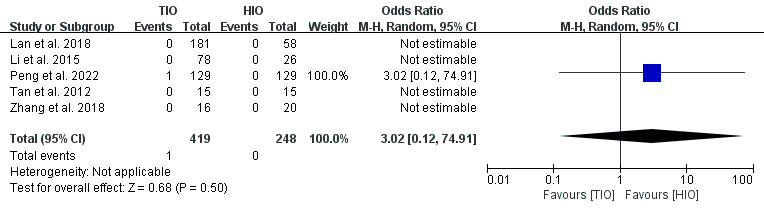


Respiratory infection


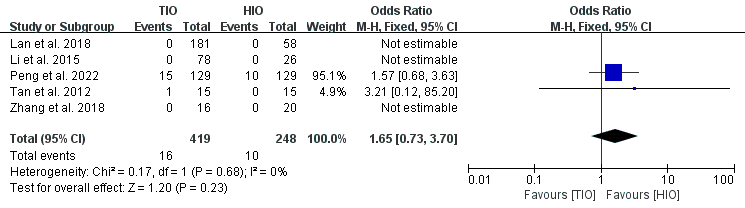


Cough


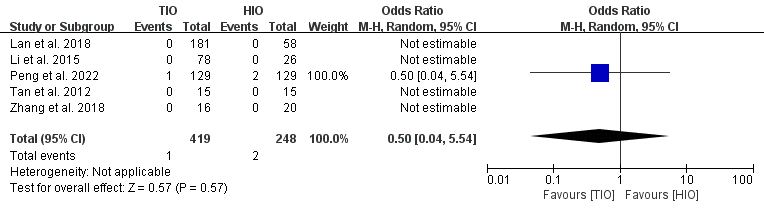


Wound infection


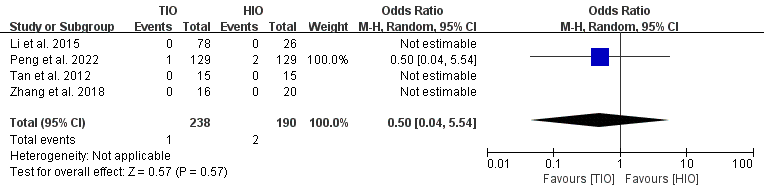


Surgical site infections


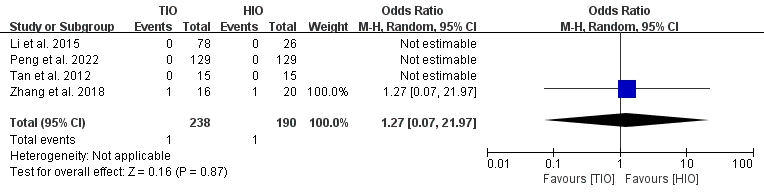

Supplement: Supplementary file 3 [file Supplementaryfile3.docx]
