# Supplementary material for: Total hepatic inflow occlusion vs. hemihepatic inflow occlusion for laparoscopic liver resection: a systematic review and meta-analysis
Source: Front Surg. 2024 Sep 26;11:1428545. doi: 10.3389/fsurg.2024.1428545 (PMC11467754; doi:10.3389/fsurg.2024.1428545)

Postoperative alanine transferase (ALT)


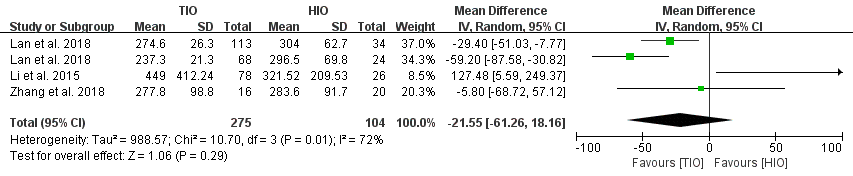


Postoperative aspartic aminotransferase (AST)


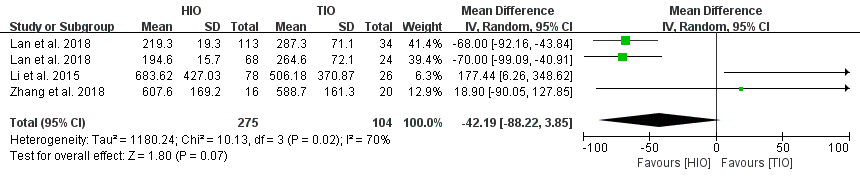


Postoperative total bilirubin (TB)


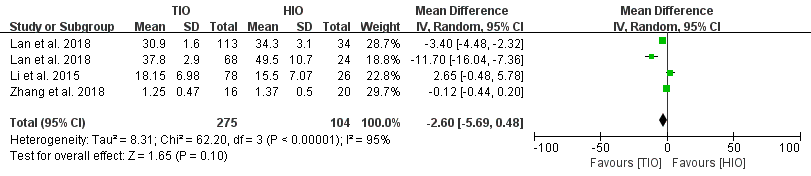


Postoperative albumin


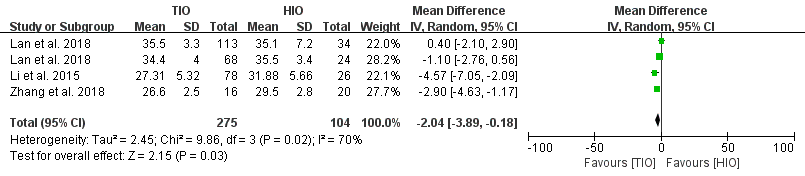

Supplement: Supplementary file 4 [file Supplementaryfile4.docx]
