# Supplementary material for: Total hepatic inflow occlusion vs. hemihepatic inflow occlusion for laparoscopic liver resection: a systematic review and meta-analysis
Source: Front Surg. 2024 Sep 26;11:1428545. doi: 10.3389/fsurg.2024.1428545 (PMC11467754; doi:10.3389/fsurg.2024.1428545)
Supplement: Supplementary file 5 [file Table1.docx]

Supplement Table 1. Basic characteristics of the enrolled studies.

|  | Year | Country | Design of study | Year of publication | Sample size | | Mean age (years) | | Sex (male/female) | |
| --- | --- | --- | --- | --- | --- | --- | --- | --- | --- | --- |
|  |  |  |  |  | TIO | HIO | TIO | HIO | TIO | HIO |
| Peng et al | 2022 | China | Randomized clinical trial | 2017-2019 | 129 | 129 | 52.4±13.0 | 52.7±12.6 | 86/ 43 | 78/ 51 |
| Zhang et al | 2018 | China | Retrospective study | 2011-2016 | 16 | 20 | 47.3±12.1 | 46.1±10.7 | 9/ 7 | 11/ 9 |
| Lan et al | 2018 | China | Retrospective study | 2015-2017 | 113 | 34 | 52.9±11.1 | 55.7±13.7 | 86/ 27 | 26/ 8 |
|  |  |  |  |  | 68 | 24 | 53.7±10.6 | 53.9±8.9 | 58/ 10 | 20/ 4 |
| Li et al | 2015 | China | Retrospective study | 2006-2015 | 78 | 26 | 44.91±8.52 | 42.92±11.47 | 24/ 54 | 4/ 22 |
| Tan et al | 2012 | China | Retrospective study | 2004-2010 | 15 | 15 | 52.0±5.5 | 49.6±6.3 | 12/ 3 | 8/ 7 |

|  | HB (g/L) | | | ALT (IU/L) | | AST (IU/L) | | TBIL (umol/L) | | ALB (g/L) | |
| --- | --- | --- | --- | --- | --- | --- | --- | --- | --- | --- | --- |
|  | TIO | HIO | | TIO | HIO | TIO | HIO | TIO | HIO | TIO | HIO |
| Peng et al | 141(69-181) | 139(85-175) | | 24.0(6.0-299.0) | 24.0(7.0-322.0) | 26.0(11.0-212.0) | 24.0(13.0-373.0) | 11.8(3.8-32.6) | 11.8(3.8-38.4) | 43.4(29.4-55.9) | 43.4(34.9-51.5) |
| Zhang et al | 128.6±21.2 | 121.8±24.8 | | 48.7±21.3 | 54.4±26.6 | 56.2±23.2 | 52.1±27.3 | 16.4±5.3 | 17.2±6.2 | 34.7±5.6 | 36.4±5.8 |
| Lan et al | / | / | | 47.1±6.9 | 44.1±5.6 | 43.0±6.1 | 42.0±5.1 | / | / | / | / |
|  | / | / | | 42.4±3.4 | 30.8±3.0 | 43.6±5.1 | 32.8±2.3 | / | / | / | / |
| Li et al | / | / | | 20.77±30.92 | 21.58±19.66 | 22.20±23.24 | 19.42±4.93 | 12.02±5.00 | 11.70±5.37 | 39.61±5.11 | 39.54±3.47 |
| Tan et al | 52.0±5.5 | | 49.6±6.3 | / | / | / | / | / | / | / | / |

|  | AFP (ng/ml) | | Child-Pugh grade (A/ B/ C) | | Malignant (HCC/ ICC/ LM) | | Benign (Hemangioma/ FNH/ Hepatolithiasis/ Others) | | | Liver cirrhosis | |
| --- | --- | --- | --- | --- | --- | --- | --- | --- | --- | --- | --- |
|  | TIO | HIO | TIO | HIO | TIO | HIO | TIO | HIO | TIO | | HIO |
| Peng et al | / | / | 129/ 0/ 0 | 129/ 0/ 0 | 70 /4/ 13 | 66/ 3/ 11 | 18/ 7/ 7/ 10 | 25/ 3/ 13/ 8 | / | | / |
| Zhang et al | / | / | 11/ 5/ 0 | 12/ 8/ 0 | 6/ 1/ 2 | 8/ 2/ 3 | 7/ 0/ 0/ 11 | 7/ 0/ 0/ 15 | 5 | | 5 |
| Lan et al | 353.0±48.0 | 274.9±81.6 | 107/ 6/ 0 | 33/ 1/ 0 | / | / | / | / | / | | / |
|  | 367.9±59.8 | 296.9±130.7 | 66/ 2/ 0 | 23/ 1/ 0 | / | / | / | / | 68 | | 24 |
| Li et al | / | / | 73/ 5/ 0 | 24/ 2/ 0 | / | / | / | / | / | | / |
| Tan et al | / | / | 10/ 3/ 2 | 13/ 2/ 0 | / | / | -/ -/ 1/ - | -/ -/ 3/ - | / | | / |

|  | Tumor diameter (cm) | | Type of resection (RH / RAH/ RPH/ LH/ II/ III/ IV/ V/ VI/ VII/ VIII/ V+VI/ VI+VII/ V+VI+VII/ VII+VIII) | | Conversion | |
| --- | --- | --- | --- | --- | --- | --- |
|  | TIO | HIO | TIO | HIO | TIO | HIO |
| Peng et al | 4.75(1.0-10.0) | 4.5(1.0-15.0) | 22/ 8/ 11/ 22/ 15/ 1/ 3/ 2/ 3/ 6/ 3/ 2/ 0/ 2/ 1 | 25/ 10/ 9/ 18/ 17/ 0/ 6/ 5/ 2/ 2/ 2/ 2/ 0/ 4/ 1 | 1 | 1 |
| Zhang et al | 4.1±2.6 | 4.3±2.8 | 0/ 0/ 0/ 0/ 0/ 0/ 0/ 0/ 0/ 7/ 0/ 0/ 8/ 0/ 0 | 0/ 0/ 0/ 0/ 0/ 0/ 0/ 0/ 0/ 11/ 0/ 0/ 9/ 0/ 0 | 1 | 1 |
| Lan et al | / | / | / | / | / | / |
|  | / | / | / | / | / | / |
| Li et al | / | / | / | / | / | / |
| Tan et al | / | / | / | / | / | / |

Notes: TIO, total hepatic inflow occlusion. HIO, hemihepatic inflow occlusion. HB, hemoglobin. ALT, alanine transferase. AST, aspartic aminotransferase. TBIL, total bilirubin. ALB, albumin. HCC, hepatocellular carcinoma. ICC, intrahepatic cholangiocarcinoma. LM, liver metastases. FNH, focal nodular hyperplasia. RH, right hemihepatectomy. RAH, right anterior hepatectomy. RPH, right posterior hepatectomy. LH, left hemihepatectomy.
